# A transcranial magnetic stimulation study of the effect of visual orientation on the putative human mirror neuron system

**DOI:** 10.3389/fnhum.2013.00679

**Published:** 2013-10-16

**Authors:** Jed D. Burgess, Sara L. Arnold, Bernadette M. Fitzgibbon, Paul B. Fitzgerald, Peter G. Enticott

**Affiliations:** Monash Alfred Psychiatry Research Centre, The Alfred and Central Clinical School, Faculty of Medicine, Nursing and Health Sciences, Monash UniversityMelbourne, VIC, Australia

**Keywords:** mirror neurons, transcranial magnetic stimulation, electromyography, associative learning, action observation, visual perspective

## Abstract

Mirror neurons are a class of motor neuron that are active during both the performance and observation of behavior, and have been implicated in interpersonal understanding. There is evidence to suggest that the mirror response is modulated by the perspective from which an action is presented (e.g., egocentric or allocentric). Most human research, however, has only examined this when presenting intransitive actions. Twenty-three healthy adult participants completed a transcranial magnetic stimulation experiment that assessed corticospinal excitability whilst viewing transitive hand gestures from both egocentric (i.e., self) and allocentric (i.e., other) viewpoints. Although action observation was associated with increases in corticospinal excitability (reflecting putative human mirror neuron activity), there was no effect of visual perspective. These findings are discussed in the context of contemporary theories of mirror neuron ontogeny, including models concerning associative learning and evolutionary adaptation.

## INTRODUCTION

Mirror neurons are a class of motor neuron that are active during both the performance and observation of behavior. Fortuitously discovered in macaque monkeys (di Pellegrino et al., 1992), an analogous “mirror neuron system” (MNS) has since been established in humans (Rizzolatti and Fabbri-Destro, 2010). From a theoretical perspective, it has been widely suggested that the MNS facilitates action understanding and other aspects of social cognition. This has been labeled the “adaptation model” of the MNS, as it suggests that mirror neurons have been selected for throughout evolution because they confer a survival and reproductive advantage (e.g., recognition of negative emotions including fear and disgust, development of interpersonal relations, child rearing, formation of complex social systems) (Gallese and Goldman, 1998; Rizzolatti et al., 2001; Meltzoff and Decety, 2003; Rizzolatti and Craighero, 2004; Bertenthal and Longo, 2007; Lepage and Theoret, 2007; Heyes, 2010). Indeed, there is evidence to suggest a link between social cognition and MNS activity among healthy individuals (Enticott et al., 2008b; Pfeifer et al., 2008; Lepage et al., 2010), while mirror neuron activity is often reduced among disorders involving impaired social cognition (e.g., autism, schizophrenia; Oberman et al., 2005; Dapretto et al., 2006; Enticott et al., 2008a,b).

Given a proposed link to interpersonal understanding, there has been some interest in the degree to which a mirror neuron response is modulated by the perspective from which an action is presented (e.g., self/egocentric vs. other/allocentric perspective). For instance, a number of transcranial magnetic stimulation (TMS) studies have investigated effects of manipulating visual orientation during the observation of hand movements. Maeda et al. (2002) showed, using intransitive movement stimuli, that simple finger and thumb movements from an egocentric perspective elicited far greater putative mirror neuron activity than movement from an allocentric perspective. Using TMS to investigate visual orientation, Alaerts et al. (2009) found that viewing right-handed intransitive actions induced a greater mirror response from an egocentric perspective, but viewing left-handed intransitive actions induced a greater mirror response from an allocentric perspective. By contrast, however, Theoret et al. (2005) did not find an effect of visual orientation (egocentric vs. allocentric) during intransitive hand action observation among their healthy control participants. Although using techniques that are generally unable to be employed in humans, Caggiano et al. (2011) found that the majority of mirror neurons in macaque F5 were “view-dependent,” responding to one of three different viewpoints.

The present study used TMS and electromyography (EMG) to investigate corticospinal excitability (CSE) whilst observing hand actions (putatively reflecting mirror neuron activity) from egocentric and allocentric perspectives. Importantly, and in contrast to previous studies, this study employed transitive action stimuli, which we have previously demonstrated is more reliably associated with a putative mirror response (Enticott et al., 2010).

## MATERIALS AND METHODS

### PARTICIPANTS

Participant demographic data is presented in **Table 1**. Twenty-three participants with no self-reported history of psychiatric or neurological illness were recruited by advertisement at Monash University and The Alfred (a teaching hospital in Melbourne, Australia). Prior to the experiment, participants were screened to ensure they met TMS safety standards (Wassermann, 1998). Participants were compensated $25 for their time and travels. The study was approved by the Alfred Hospital Ethics Committee and the Monash University Human Research Ethics Committee. Participants provided written informed consent prior to participation in the study.

**Table 1:** Participant demographics

*n*	23
Gender (M:F)	13:10
Age (Years)	23.09 (3.75)
Age range (Years)	18–31
Formal education (Years)	15.91 (1.41)
Handedness (L:R)^a^	4–19

a Assessed using the Edinburgh Handeness Inventory (Oldfield, 1971).

### MATERIALS

Short video clips depicting either a static hand or a hand grasping a mug were used to measure putative MNS activity. We elected to use only a static hand control as our previous research has indicated that additional control stimuli (e.g., static hand with object, pantomimed grasp) do not significantly modulate CSE (Enticott et al., 2010). Stimuli were presented from both egocentric (i.e., self) and allocentric (i.e., other) perspectives.

**FIGURE 1:**
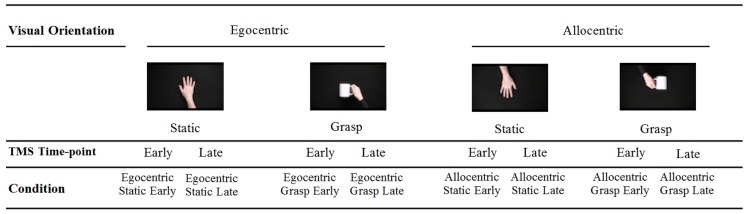
Stimuli presented during TMS administration.

Screen shots of the videos are displayed in **Figure 1.** Participants were shown two blocks of videos each consisting of 40 video clips (80 in total, 20 of each condition: static egocentric, active egocentric, static allocentric, active allocentric). Each block of videos ran for 5 m 05 s, and there was a short break (2–3 m) between blocks. All clips were 4 s in length, appearing in a quasi- randomized sequence with a 2 s gap (black screen) between each.

### PROCEDURE

Using a 70 mm figure-of-eight coil, single pulse TMS was administered to the scalp at the left primary motor cortex (M1; scalp location resulting in largest motor-evoked potential from the right first dorsal interosseous, FDI). Resting motor threshold (RMT) was defined as the minimum stimulation intensity that evoked a peak-to-peak MEP of >50 μV in at least three out of five consecutive trials.

MEP data were recorded from right FDI via EMG using self-adhesive electrodes. This signal was amplified by the PowerLab/4SP (AD instruments, Colorado Springs, CO, USA) and sampled via a CED Micro 1401 mk II analog-to-digital converting unit (Cambridge Electronic Design, Cambridge, UK). Participants viewed the video presentations seated 120 cm away from a 56 cm widescreen LCD monitor positioned at eyelevel in a comfortable reclining chair.

Participants were administered a TMS pulse (120% RMT) during each video clip and their MEP was recorded. A light sensor placed on the top right-hand corner of the LCD monitor was used to control the timing of the TMS pulse. In order to activate the light sensor, a 4 cm × 4 cm white-square was embedded within the clips [i.e., in the top right hand corner of the screen for a period of 1 frame (40 ms)] at two time intervals (i.e., “early” at the 2 s mark and “late” at the 3 s mark). Two time-points were used to minimize anticipation of the TMS pulse. This was also in accord with previous research illustrating that MEP amplitude corresponds significantly to finger aperture of grasping actions (Gangitano et al., 2001, 2004), and that MEP is greatest 60–90 ms after the onset of a finger movement (Lepage et al., 2010). The embedded white square time-locked the TMS pulse to each video clip through a 5 V TTL pulse delivered via a BNC connector. A second trigger was sent from the TMS stimulator upon activation of the pulse to the EMG device to initiate MEP recording.

### DATA ANALYSIS

Participants’ median CSE values were then indexed to provide a ratio of change between the “grasp” versus “static” conditions (i.e., median CSE amplitude for “grasp” conditions/median CSE amplitude for “static” conditions × 100; be they “early” or “late” respectively) This is referred to as the MEP-Ratio. This is a common approach whereby an MEP-Ratio above 100% reflects putative mirror neuron activity (Enticott et al., 2012a). The use of median (rather than mean) valuesis also consistent with our previous research (e.g., Enticott et al., 2010, 2012a,b), and is intended to minimize the influence of transient increases in CSE than can occur during the early stages of a TMS experiment (Schmidt et al., 2009).

The distributions of MEP-Ratio datawere examined for extreme outliers (±3 standard deviations from the mean) in each condition. One participant was omitted due to consistently outlying data. Based on recommendations within the statistical literature (e.g., Tabachnick and Fidell, 1996), the remaining extreme outliers were reduced to one value above the next highest data point to minimize their influence. There were two extreme outliers in the Egocentric-Early condition, one in the Allocentric-Early condition and three in the Allocentric-Late condition. Finally, to satisfy the assumption of normality, the square root of the MEP-Ratio was derived and used for analysis.

We conducted a 2 (timepoint: early vs. late) × 2 (viewpoint: egocentric vs. allocentric) repeated-measures ANOVA to compare the MEP-Ratio across the four action observation conditions (i.e., egocentric-early, egocentric-late, allocentric-early and allocentric-late). For all analyses, sphericity was violated and a Greenhouse-Geisser correction was used.

## RESULTS

**FIGURE 2:**
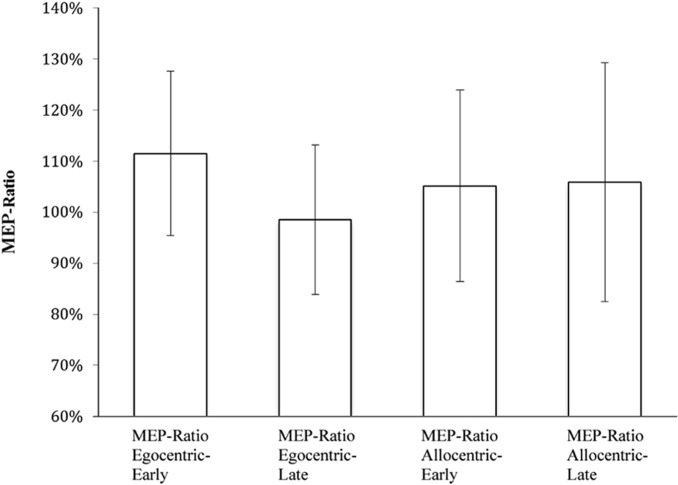
MEP-Ratios for video presentation conditions.

**FIGURE 3:**
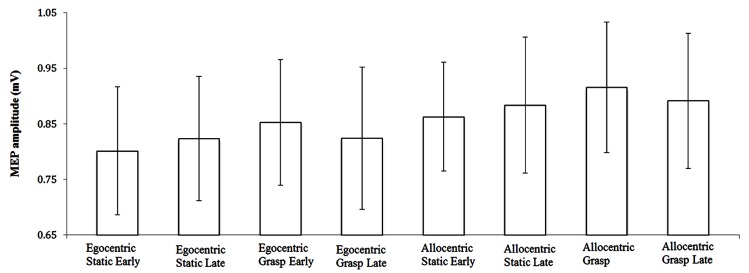
Raw MEP amplitude (±SE) for video presentation conditions.

MEP-Ratio results are presented in **Figure 2**, and raw MEP amplitudes (although not subject to inferential analyses) are presented in **Figure 3**. A one-sample *t*-test for action observation conditions combined revealed a significant increase above 100% (*M* = 105.98, *SD* = 13.60), *t*(22) = 2.11, *p* = 0.047, suggesting that, consistent with previous research, action observation produced the expected increase in CSE above static hand observation. There was no significant interaction between viewpoint and time-point, *F*(1,22) = 2.43, *p* = 0.133, $\eta_{p}^{2}$ = 0.10. Similarly, there was no difference in CSE between the egocentric and allocentric viewpoints, *F*(1,22) = 0.73, *p* = 0.403, $\eta_{p}^{2}$ = 0.03, nor was there a difference between the early and late TMS pulse time-points, *F*(1,22) = 2.61, *p* = 0.120, $\eta_{p}^{2}$ = 0.11.

While there was an overall MEP-Ratio increase above 100%, this was not uniformly found across the four individual conditions (egocentric-early: *t*[22] = 2.26, *p* = 0.034; egocentric-late: *t*[22] = 0.06, *p* = 0.950; allocentric-early: *t*[22] = 0.78, *p* = 0.442; allocentric-late: *t*[22] = 1.16, *p* = 0.260). Accordingly, it might be argued that this fails to provide sufficient evidence of a mirror response to the stimuli across all conditions. In an attempt to address this concern, we conducted a subsequent analysis involving only those 15 participants who displayed, overall, a facilitation effect (i.e., mean MEP-Ratio > 100%). The range of mean overall MEP-Ratios for this subgroup was 103–138% (compared with 85–99% for those excluded from this analysis), while 14 of the 15 participants in the subgroup also displayed a MEP-Ratio of >110% in at least one of the two viewpoint conditions. This was justified on the theoretical basis of the paradigm (i.e., a score >100% indicating a mirror neuron response), and was intended to determine whether a sample that show consistent facilitation effects would reveal the same pattern of results as the broader sample.

Based on our findings, which revealed no effect of time-point for either the full sample or subgroup (see below), the two time points were averaged for each condition. One-sample *t*-tests indicated that these participants displayed a significant increase in MEP-Ratio for egocentric (*M* = 111.60%, *SD* = 14.14), *t*(14) = 3.18, *p* = 0.007, and a near significant increase for allocentric (*M* = 113.71%, *SD* = 25.19), *t*(14) = 2.11, *p* = 0.053.

A subsequent 2 (timepoint: early vs. late) × 2 (viewpoint: egocentric vs. allocentric) repeated-measures ANOVA with this subgroup revealed no effect of viewpoint, *F*(1,14) = 0.01, *p* = 0.928, $\eta_{p}^{2}$ = 0.001, or timepoint, *F*(1,14) = 0.26, *p* = 0.617, $\eta_{p}^{2}$ = 0.02, and no interaction effect, *F*(1,14) = 0.25, *p* = 0.619, $\eta_{p}^{2}$ = 0.02.

## DISCUSSION

The current study was designed to examine whether there were differences in the putative mirror neuron response when viewing the same action from different visual perspectives. There did not appear to be an effect of visual orientation on MEP-Ratio, our measure of putative mirror neuron activity. Although a failure to demonstrate consistent facilitation effects means that we must be careful in interpreting these data, these findings are inconsistent with some previous studies assessing the effect of visual orientation on a TMS-induced mirror neuron response (e.g., Maeda et al., 2002; Alaerts et al., 2009). There are, however, a number of differences between these studies and ours, including the use of transitive stimuli in the current study (which we have demonstrated is more reliably associated with corticospinal facilitation; Enticott et al., 2010). It may transpire, for example, that different mechanisms underlie the mirror response to transitive and intransitive movements (for example, different populations of mirror neurons that result in differences in motor CSE), similar to what was found among macaques by Caggiano et al. (2011). It should be noted, however, that Kraskov et al. (2009) showed, among macaques, that 73% of mirror neurons that responded to a transitive action also responded to an equivalent intransitive action. Alternatively, motor CSE during transitive movements may result from a combination of mirror neuron (responsive to biological motion) and canonical neuron (responsive to motion but also objects) activation, again raising the possibility of a different pattern of motor CSE.

From a theoretical perspective, mirror neurons are often seen from an evolutionary perspective, where a genetic component is necessarily assumed, and often a relatively minimal role is attributed to sensorimotor experience. By contrast, a more recent model suggests that mirror neurons are not of evolutionary importance, but rather a product of associative learning that takes place during sensorimotor processing (e.g., visual and motor activity, such as during the observation of one’s own hand movement; Cook et al., 2010; Cooper et al., 2013). The strongest evidence for such a model demonstrates that relatively limited sensorimotor training can significantly modulate putative human mirror neuron activity (Haslinger et al., 2005; Catmur et al., 2007; Capa et al., 2011; Wiggett et al., 2011). Proponents of this “association model” suggest that mirror neurons have not evolved to facilitate action understanding (Heyes, 2010; Cook et al., in press).

The association models might predict that mirror neuron activity should be enhanced for those associations that are more strongly established (Heyes, 2010). Similarly, the more an action stimulus represents a strongly held association, the greater the mirror neuron response. One example of association that would produce mirror neurons involves hand-eye coordination. Typically, hand actions involving affordances (e.g., grasping a mug) are visually monitored by the individual performing the action. This ensures simultaneous activation of both visual and motor neurons, which allows the formation of an association where, eventually, activation of visual neurons is sufficient to produce activation of some motor neurons (i.e., mirror neurons; Casile et al., 2011). For instance, hand-eye coordination is clearly embedded within an egocentric (i.e., self) viewpoint. Thus, under the association model, it could be conceived that actions viewed from an egocentric perspective should elicit a more pronounced mirror response, as this perspective is more common for synchronous visual-motor activity and therefore has stronger associations.

Although these data seem thereforeinconsistent with this aspect of the association model, it is not clear whether they are necessarily consistent with the adaptation model. As noted, an adaptation account maintains that the MNS has evolved to serve the needs of action understanding and related social cognitive abilities. Thus, it might be argued that any system designed to facilitate this behavioral understanding should process visual stimuli comparably across all orientations, as the essential meaning to be derived is the same. In this respect these results might be seen as compatible with no preference for specific perspectives, as is clearly the case here. Alternatively, it might be argued that the adaptation model should favor the allocentric perspective in order to understand others’ behavior, which is inconsistent with the current findings. Reconciliation of the adaptation model with previous research illustrating training effects (Haslinger et al., 2005; Catmur et al., 2007; Capa et al., 2011; Wiggett et al., 2011) is similarly difficult.

There is, however, an alternative interpretation within the associative learning model that could account for the current findings. Proponent of this theory, which is based on the associative learning literature (including the Rescorla-Wagner model of conditioning, which concerns the strength of prediction for one cell firing together with another; Cooper et al., 2013), might suggest that there are ceiling effects to the formation of visuomotor associations when events are no longer novel or surprising. By adulthood, there may have been sufficient experience to allow visuomotor associations across the various visual perspectives. While this will require further research and theoretical development, under this model it is conceivable that we should see equivalent mirror neuron activation across differing perspectives.

There are several limitations to this research. Perhaps most importantly, there was a great deal of variability across our data, and not all of the individual conditions displayed a significant facilitation effect. Although the results held when investigating a subset of participants who displayed facilitation, it remains that we must interpret these data cautiously. These data ultimately do not allow us to draw firm conclusions about the influence of visual perspective at this point. Another limitation concerns the ecological validity of the video presentations. In order to maintain experimental control, grasping actions needed to remain consistent throughout the video clips. Due to technical constraints, the most tenable solution was to film the stimuli from above, appearing egocentrically orientated. This camera setup allowed for an allocentric orientation to be created by flipping and rotating the original clips. While all effort was made to maintain proper ecological validity (including preventing the mug from appearing upside down), it is conceivable that some participants may have had concerns with the realism of stimuli from the allocentric orientation, particularly with the perceived orientation of the mug. Nevertheless, our approach was consistent with other studies of mirror neurons and visual perspective (Maeda et al., 2002; Theoret et al., 2005; Alaerts et al., 2009). From a theoretical perspective, the association model would predict an increased mirror response with a stimulus that more closely approximates stored associations; accordingly, even if not a true allocentric representation, the stimuli used in the current study provide such an approximation. We will, however, attempt a more ecologically valid methodology in any subsequent research (e.g., simultaneous filming of a single action from different perspectives).

In summary, when examining the effect of egocentric and allocentric orientated goal-directed visual stimuli, measures of MEP-Ratio (i.e., putatively reflecting mirror neuron activity) did not appear to differ across perspectives. It is unclear, however, whether or not these findings are consistent with current models of mirror neuron ontogeny, and this area will require further theoretical and empirical investigation.

## Conflict of Interest Statement

The authors declare that the research was conducted in the absence of any commercial or financial relationships that could be construed as a potential conflict of interest.
